# Serum Amyloid A Promotes E-Selectin Expression via Toll-Like Receptor 2 in Human Aortic Endothelial Cells

**DOI:** 10.1155/2016/7150509

**Published:** 2016-10-05

**Authors:** Eisaku Nishida, Makoto Aino, Shu-ichiro Kobayashi, Kosuke Okada, Tasuku Ohno, Takeshi Kikuchi, Jun-ichiro Hayashi, Genta Yamamoto, Yoshiaki Hasegawa, Akio Mitani

**Affiliations:** ^1^Department of Periodontology, School of Dentistry, Aichi Gakuin University, 2-11 Sumemori-dori, Chikusa-ku, Nagoya, Aichi 464-8651, Japan; ^2^Department of Microbiology, School of Dentistry, Aichi Gakuin University, 1-100 Kusumoto-cho, Chikusa-ku, Nagoya, Aichi 464-8650, Japan

## Abstract

Periodontitis is a chronic inflammatory disease that affects the periodontium. Recent studies suggest an association between periodontal and cardiovascular diseases. However, the detailed molecular mechanism is unknown. A previous study has demonstrated that experimental periodontitis induces serum amyloid A (SAA) in the liver and peripheral blood of ApoE-deficient mice as an atherosclerosis model. SAA is an acute-phase protein that affects systemic inflammation. The aim of this study is to investigate the atherosclerosis-onset mechanism using human aortic endothelial cells (HAECs) stimulated by SAA* in vitro*. Atherosclerosis PCR array and qPCR analyses showed upregulation of adhesion molecules such as intercellular adhesion molecule-1, vascular cell adhesion molecule-1, and E-selectin in HAECs upon SAA stimulation. In addition, the results demonstrated that Toll-like receptor, TLR2, could serve as an important receptor of SAA in HAECs. Furthermore, small interfering RNA (siRNA) against TLR2 inhibited the upregulation of adhesion molecules in HAECs stimulated by SAA. Our results suggest that SAA stimulates the expression of adhesion molecules via TLR2. SAA could be an important molecule for atherosclerosis induced by periodontal disease.

## 1. Introduction

Periodontitis, one of the most common diseases in humans and is an infectious disease that can result in inflammatory destruction of the periodontium (alveolar bone, cementum, periodontal ligament, and gingiva) [1]. Periodontal disease has been recognized as a risk or contributing factor for systemic diseases including atherosclerotic vascular disease, diabetes mellitus, rheumatoid arthritis, Alzheimer's disease, and cancer [2–9].

The prevalence and incidence of coronary heart disease are significantly increased in patients with periodontitis, indicating that periodontal disease independently predicts coronary heart disease [10]. Recent systematic reviews and meta-analysis of observational studies support an association between periodontal and atherosclerotic vascular diseases, which is independent of known confounders, but a causal relationship is not yet established [11, 12].

Serum amyloid A (SAA), an acute-phase protein, is markedly upregulated in response to infection and during chronic inflammation [13–15]. SAA stimulates vascular cells to express cytokines, chemokines, adhesion molecules, and matrix metalloproteinases [16], which are linked to the development of atherosclerosis. In addition, high levels of SAA in peripheral blood are significantly associated with periodontitis, and SAA levels are decreased in patients with periodontitis after periodontal therapy [17, 18].

Recently, evidence of the possible link between periodontitis and atherosclerosis has increased, and the possible association and causality are being investigated [19, 20]. Hujoel et al. reported an important general trend towards periodontal treatment-induced inhibition of systemic inflammation and improvement in noninvasive markers of atherosclerosis and endothelial function [19]. However, the detailed molecular mechanism for periodontitis-induced atherosclerosis is unknown.

The aim of this study is to investigate the atherosclerosis-onset mechanism using human aortic endothelial cells (HAECs) stimulated by SAA* in vitro*. Here, we demonstrate that stimulation of HAECs with SAA results in the induction of adhesion molecules, which may be caused via Toll-like receptor, TLR2.

## 2. Materials and Methods

### 2.1. Cell Culture and SAA Treatment

HAECs were purchased from Lonza (CC-2535; Tokyo, Japan) and used in all experiments. The HAECs were cultured in Endothelial Cell Growth Medium 2 (EBM-2) medium supplied with the EBM-2 bullet kit (Lonza, Tokyu, Japan) at 37°C with 5% CO_2_. Subconfluent passage 6 cells were used in all experiments. HAECs were plated at 1.5 × 10^5^ cells/well in 6-well plates and cultured to subconfluence. The cells were then treated with 1.5 *μ*g/mL recombinant human SAA (PeproTech, Rocky Hill, NJ) for 0, 1, 3 and 6 h.

### 2.2. PCR Array Analysis

Total RNA was extracted from HAECs using NucleoSpin RNA II (Macherey-Nagel, Diiren, Germany). First-strand cDNA synthesis was performed using RT^2^ First Strand Kit (Qiagen, Tokyo, Japan) following the manufacturer's instructions. The Human Atherosclerosis RT^2^ Profiler*™* PCR Array (PAHS-038Z) (Qiagen, Tokyo, Japan) was applied to an ABI 7000 Real-Time PCR System (Applied Biosystems, Foster City, CA). The RT^2^ Profiler*™* PCR Array for Human Atherosclerosis contains 84 genes for responses to stress, apoptosis, blood coagulation and circulation, adhesion molecules, extracellular molecules, lipid transport and metabolism, and cell growth and proliferation. In addition, the array contains five wells for various housekeeping genes, a genomic DNA contamination control, three replicate reverse transcription controls, and three replicate positive PCR controls. Data analyses were performed using web-based analysis software (http://pcrdataanalysis.sabiosciences.com/pcr/arrayanalysis.php).

### 2.3. qPCR Analysis

cDNAs were synthesized from 1 *μ*g total RNA using Revetra Ace qPCR Master Mix (Toyobo, Osaka, Japan). qPCR was performed with THUNDERBIRD SYBR qPCR Mix (Toyobo, Osaka, Japan) and TaqMan Universal Master Mix II (Applied Biosystems, Foster City, CA) using the ABI 7000 Real-Time PCR System. SYBR Green primers (SELS, ABCA1, ABCA7, SCARB1, CD36, TLR2, TLR4, CST3, FPR2, AGER, and GAPDH) were designed by Primer3 software (http://bioinfo.ut.ee/primer3-0.4.0/) (Table 1). The expression of each gene was normalized to the GAPDH level (SYBR Green). TaqMan primers (TLR2, Hs01872448_s1; MYD88, Hs01573837_g1; NFKB1m Hs00765730_m1; tumor necrosis factor-*α* (TNF-*α*), Hs01113624_g1; E-selectin (SELE), Hs00950401_m1) were purchased from Life Technology (Tokyo, Japan). The expression of each gene was normalized to the 18S rRNA (4319413E, Applied Biosystems, Foster City, CA) level (TaqMan). The fold change between mRNA expression levels was determined as follows: fold change = 2^−ΔΔCt^, where ΔΔCt = (Ct_target_ − Ct_18S rRNA_) treated  group − (Ct_target_ − Ct_18S rRNA_) control group (Ct, cycle threshold).

### 2.4. RNA Interference

HAECs were transfected with TLR2 Silencer Select Pre-Designed Small Interfering RNA (siRNA, ASO0ZS41; Life Technology, Tokyo, Japan) using Lipofectamine 2000 transfection reagent (Life Technology, Tokyo, Japan) according to the manufacturer's instructions. HAECs (1.5 × 10^5^ cells/well) were seeded in 6-well plates at 24 h before transfection. Then, the cells were transfected with siRNA against TLR2 at a final concentration of 10 nM. Cells were incubated with siRNA in EBM-2 medium for 24 h. Then, the medium was replaced with fresh medium, and the cells were incubated for another 48 h. HAECs were then stimulated with SAA before harvesting. TLR2 knockdown was verified by qPCR. Control siRNA was purchased from Life Technology.

### 2.5. Western Blotting

HAECs were lysed for 30 min on ice in lysis buffer (Bio-Rad, Richmond, CA) with 1 *μ*g/mL protease inhibitors. Cell debris was removed by centrifugation (14,000 ×g, 4°C, 15 min), and supernatants were collected and stored at −80°C until use. Total protein samples (30 *μ*g each) were boiled for 5 min, resolved by 10% sodium dodecyl sulfate (SDS) polyacrylamide gel electrophoresis in Tris/glycine/SDS buffer (25 mM Tris, 250 mM glycine, and 0.1% SDS), and blotted onto transfer membranes (Bio-Rad, Richmond, CA) (100 V, 1.5 h, 4°C). After blocking for 2 h in TBST (20 mM Tris-HCl, 150 mM NaCl, and 0.1% Tween 20) containing 5% dry nonfat milk, the membranes were washed three times in TBST and probed for 18 h at 4°C with the anti-TLR2 monoclonal antibody TL2.1 (final concentration: 1 *μ*g/mL; Abcam, Cambridge, MA) and anti-*β* actin monoclonal antibody (1 : 1000 dilution; Cell Signaling Technology, Beverly, MA) in TBST. After three washes in TBST, the membranes were incubated with horseradish-peroxidase-conjugated goat anti-mouse IgG (1 : 2000 dilution; Cell Signaling Technology, Beverly, MA) and then washed five times in TBST. Protein bands were detected using ECL reagents (GE Healthcare, Waukesha, WI) according to the manufacturer's instructions.

### 2.6. Statistical Analysis

Statistical analyses were performed using SPSS software v. 15.0 J for Windows (SPSS Inc., Chicago, IL). Data are expressed as the mean ± standard deviation. Student's* t*-test was used for comparisons. Significance was accepted at *p* < 0.05.

## 3. Results

### 3.1. SAA Induces Adhesion Molecules in HAECs

**Figure 1:**
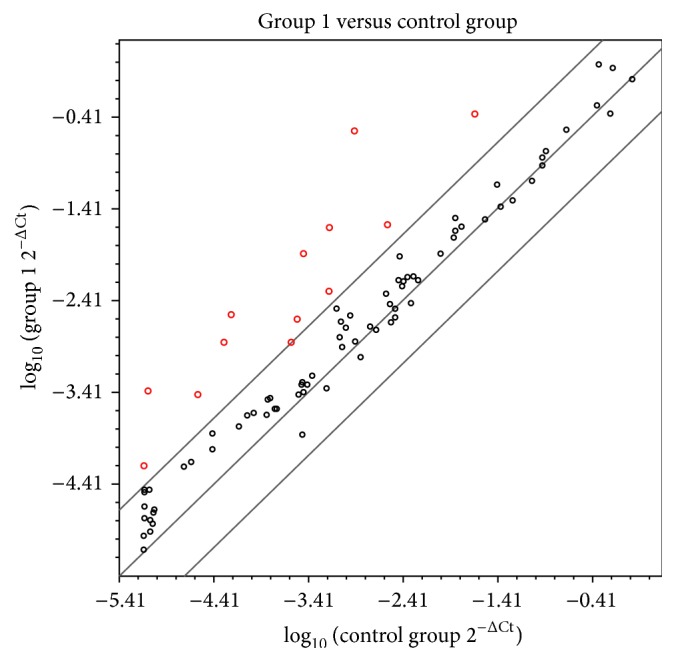
Gene screening by the RT^2^ Profiler*™* PCR Array for Human Atherosclerosis in SAA-stimulated HAECs. A total of 84 atherosclerosis-related genes were analyzed using the RT^2^ Profiler*™* PCR Array (*n* = 1 per group). Thirteen genes were identified with a more than a 5-fold change in expression between human aortic endothelial cells (HAECs) and HAECs treated with SAA for 6 h (red circles). *x*-axis: Control Group, HAECs; *y*-axis: Group 1, HAECs treated with SAA.

To explore atherosclerosis-related genes in SAA-stimulated HAECs, we used a Human Atherosclerosis RT^2^ Profiler*™* PCR Array (Figure 1). The comparison between HAECs at 0 h and 6 h after stimulation with SAA indicated specific up-regulation (>5-fold) of 13 genes including BIRC3 (baculoviral IAP repeat containing 3), CCL2 (chemokine (C-C motif) ligand 2), CCL5 [chemokine (C-C motif) ligand 5], CCR2 [chemokine (C-C motif) receptor 2], CSF2 [colony-stimulating factor 2 (granulocyte-macrophage)], FGA (fibrinogen alpha chain), ICAM1 (intercellular adhesion molecule-1), IL1A (interleukin 1, alpha), LIF [leukemia inhibitory factor (cholinergic differentiation factor)], NFKB1 (nuclear factor of kappa light polypeptide gene enhancer in B-cells 1), SELE, TNFAIP3 (tumor necrosis factor, alpha-induced protein 3), and VCAM1 (vascular cell adhesion molecule-1) (Figure 1 and Table 2). Thus, adhesion molecules such as ICAM1, VCAM1, and SELE may be upregulated in HAECs under inflammatory conditions. Among these molecules, expression of the SELE gene was remarkable (232-fold). Therefore, SAA might have an important role in the leukocyte adhesion cascade.

### 3.2. TLR2 Is Upregulated by SAA among Receptor Molecules in HAECs

**Figure 2:**
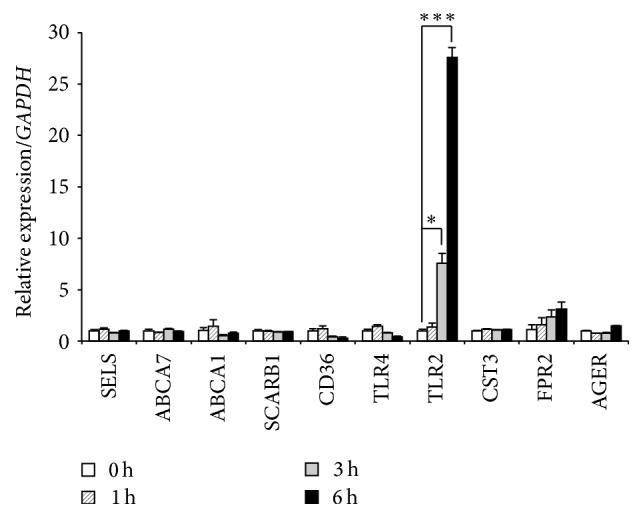
Screening of SAA receptors in HAECs. qPCR analysis of 10 genes that encode known SAA receptors was conducted. HAECs were treated with recombinant human SAA, and total RNA was extracted at 0, 1, 3, and 6 h. Among the expression levels of SAA receptors, TLR2 mRNA expression induced by SAA was the highest and significantly higher than that in unstimulated HAECs. Each experiment was performed in triplicate. Values were the mean ± SD. ^*∗*^ *p* < 0.05, ^*∗∗∗*^ *p* < 0.001.

To identify genes related to the leukocyte adhesion cascade, we screened SAA receptors that were highly expressed in HAECs during SAA stimulation (Figure 2). SAA receptors, such as SELS (glucose homeostasis and ER stress), ABCA1, ABCA7, SCARB1 (cholesterol efflux), CD36, TLR2, TLR4, CST3 (inflammatory signaling), FPR2 (chemotaxis and immune cell activation), and AGER (amyloidosis), have been reported previously [21]. Among the candidate receptors, TLR2 mRNA expression was significantly induced by SAA in HAECs, indicating that TLR2 could serve as an important receptor for SAA. Thus, SAA may stimulate the expression of adhesion molecules via TLR2.

### 3.3. SAA Induces TLR2 and Its Related Genes following the Leukocyte Adhesion Cascade

**Figure 3:**
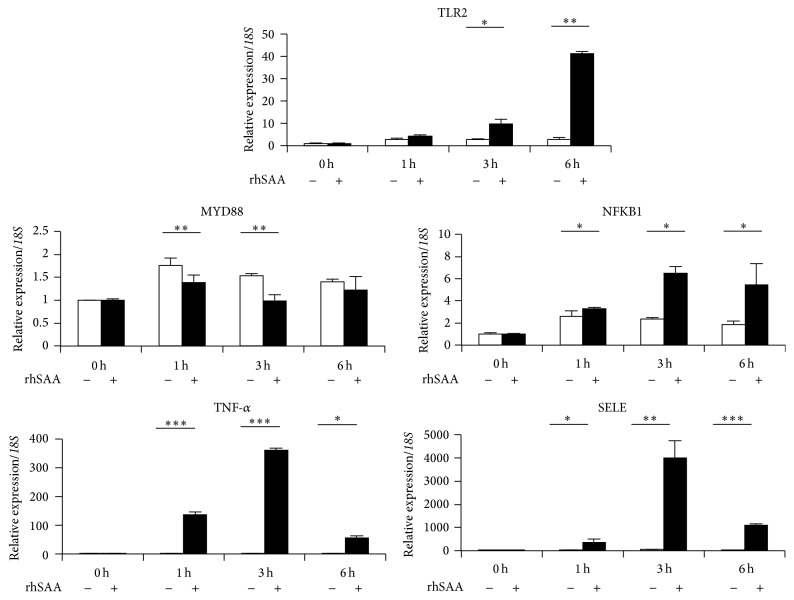
Expression pattern of TLR and selectin cascade genes in HAECs stimulated by SAA. Expression levels of TLR2, MYD88, NFKB1, TNF-*α*, and SELE mRNAs were examined at 0, 1, 3, and 6 h in HAECs stimulated by SAA. The mRNA expression of TLR2 was induced by SAA (closed column) in a time-dependent manner. Furthermore, the mRNA expression of NFKB1, TNF-*α*, and SELE was significantly upregulated by SAA (closed column) compared with the control (open column). Data represent the mean ± SD of four independent experiments. Each experiment was performed in triplicate. ^*∗*^ *p* < 0.05, ^*∗∗*^ *p* < 0.01, and ^*∗∗∗*^ *p* < 0.001. rhSAA: recombinant human SAA.

To investigate the leukocyte adhesion cascade induced by SAA, mRNA expression was examined at 0, 1, 3, and 6 h after SAA stimulation in HAECs (Figure 3). TLR2 mRNA expression was upregulated in a time-dependent manner. Furthermore, the mRNA expression of NFKB1, TNF-*α*, and SELE was significantly upregulated at 3 h after SAA stimulation. The mRNA expression of these genes was significantly higher in SAA-stimulated HAECs compared with unstimulated HAECs. However, only the mRNA expression of MYD88 was lower in SAA-stimulated HAECs compared with unstimulated HAECs. These results indicate that SAA affects downstream of TLR signaling and induces leukocyte adhesion cascade-related genes.

### 3.4. Knockdown of TLR2 Affects the Expression of SELE through the Leukocyte Adhesion Cascade

**Figure 4:**
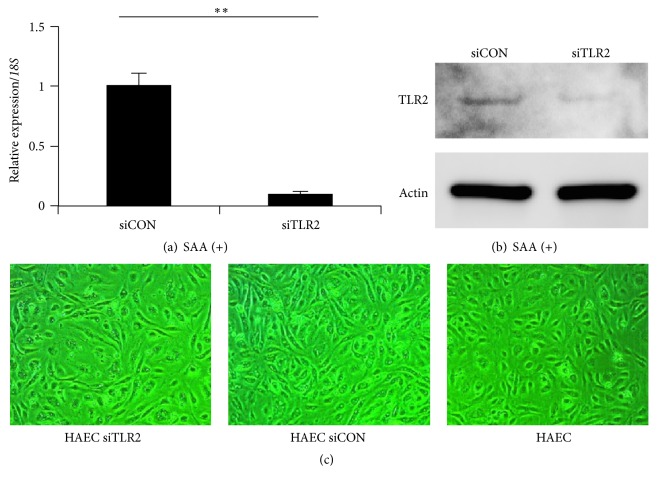
Effect of TLR2 siRNA on TLR2 expression and morphology in HAECs. The effect of TLR2 siRNA in HAECs was determined by qPCR analysis (a), western blotting (b), and observations of cell morphology (c). (a, b) mRNA and protein levels of TLR2 in HAECs were dramatically decreased by siRNA treatment for 6 h. (c) siTLR2 had no significant effect on the cell morphology of HAECs. Representative data of three independent mRNA experiments are shown. Each mRNA experiment was performed in triplicate. Values are the mean ± SD. ^*∗∗*^ *p* < 0.01. siCON: control siRNA; siTLR2: TLR2 siRNA.

**Figure 5:**
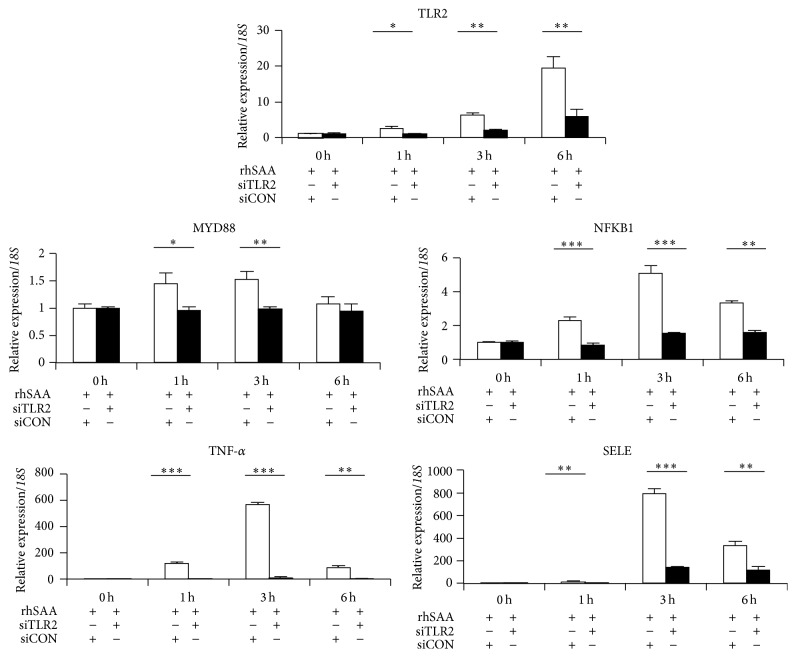
Effect of TLR2 siRNA on the expression of TLR and selectin cascade genes in HAECs. The mRNA expression levels of TLR2, MYD88, NFKB1, TNF-*α*, and SELE were examined in HAECs treated with TLR2 siRNA (closed column). After SAA stimulation for 0, 1, 3, and 6 h, the mRNA expression of all genes was dramatically decreased compared with the control (open column). Data represent the mean ± SD of four independent experiments. Each experiment was performed in triplicate. ^*∗*^ *p* < 0.05, ^*∗∗*^ *p* < 0.01, and ^*∗∗∗*^ *p* < 0.001. rhSAA: recombinant human SAA; siCON: control siRNA; siTLR2: TLR2 siRNA.

To determine the contribution of TLR2 as a SAA receptor for SELE expression, TLR2 was knocked down by transfection of siRNA. We confirmed specific knockdown of TLR2 by qPCR and western blotting (Figures 4(a) and 4(b)). No difference in cell morphologies was found in HAECs transfected with TLR2 siRNA (Figure 4(c)). Silencing of TLR2 using siRNA resulted in a significant reduction in the expression of SELE mRNA compared with cells transfected with control siRNA under SAA stimulation (Figure 5). In addition, mRNA expression of NFKB1 and TNF-*α* under SAA stimulation was abolished by silencing TLR2.

## 4. Discussion

In this study, we found that SAA induced adhesion molecules, especially SELE, and TLR2 may be a critical receptor for this process in HAECs. To our knowledge, this is the first report indicating that TLR2 plays a role in HAECs as a SAA receptor to induce adhesion molecules, which might be involved in the onset of atherosclerosis.

The mRNA expression of adhesion molecules, including ICAM1, VCAM1, and SELE, was dramatically upregulated (>5-fold) by SAA stimulation in HAECs (Figure 1). E-selectin is important for the initial rolling interactions of neutrophils, monocytes, natural killer cells, and a subset of memory T cells in the inflamed endothelium [22, 23]. Leukocyte arrest during rolling is mediated by binding of leukocyte integrins to immunoglobulin superfamily members, such as VCAM-1 and ICAM-1, which are expressed by endothelial cells [24].

The initial step of atherosclerosis includes adhesion of peripheral blood leukocytes to activate the endothelial monolayer, directed migration of the bound leukocytes into the intima, and maturation of monocytes into macrophages and their uptake of lipids, yielding foam cells [25]. Multiple members of selectin, integrin, and immunoglobulin gene families in the process of initial attachment (rolling), stable adhesion (arrest), spreading, and ultimately diapedesis are sequentially involved in the onset of atherosclerosis [25]. Based on the PCR array results, SAA induces adhesion molecules and thus may trigger arteriosclerosis onset.

Several SAA receptors have been reported previously [21]. Among them, the expression of TLR2 was dramatically increased (about 30-fold) during SAA stimulation (Figure 2). We confirmed upregulation of TLR2 at both mRNA and protein levels by SAA stimulation, and SAA increased the expression of SELE in conjunction with the leukocyte adhesion cascade in HAECs. Endothelial cells normally express TLR2 at a very low level [26]. Thus, our results suggest that TLR2 may be an important receptor for SAA to induce adhesion molecules in HAECs.

Based on qPCR analysis of downstream molecules in TLR2 signaling (Figure 3), SAA stimulation had a remarkable influence on their expression. In particular, expression of the leukocyte cascade (i.e., NFKB1 and TNF-*α*) was upregulated following the induction of TLR2 by SAA stimulation. In addition, analysis of HAECs transfected with siRNA against TLR2 showed downregulation of the expression of the leukocyte cascade (NFKB1 and TNF-*α*) and SELE in a time-dependent manner (Figure 5).

MyD88 has a critical role in signaling via TLR2. After stimulation, TLR recruits IL-1R-associated kinase via adaptor MyD88 and induces activation of mitogen-activated protein kinases and nuclear factor-*κ*B (NF-*κ*B) [27]. NF-*κ*B activation induces proinflammatory cytokines, including TNF-*α* and chemokines [28]. Furthermore, in human endothelial cells, adhesion molecules such as E-selectin are induced by NF-*κ*B and TNF-*α* [29]. Nevertheless, in the current study, mRNA expression of MYD88 was decreased in SAA-stimulated HAECs, and the mRNA expression of NFKB1, TNF-*α*, and SELE was upregulated. We speculate that SAA may induce NF-*κ*B, TNF-*α*, and E-selectin, in part, through a MyD88-independent pathway via TLR2. Indeed, Nilsen et al. have recently reported a novel function of TRAM/TRIF in TLR2-mediated signal transduction [30]. However, further studies are needed to investigate the detailed mechanism of MyD88-dependent or MyD88-independent signaling pathways in HAECs treated with SAA.

## 5. Conclusions

**Figure 6:**
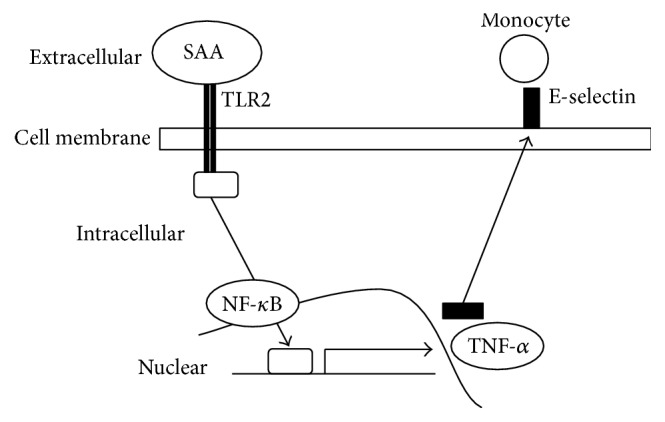
Summary of the possible mechanism for induction of E-selectin by SAA via TLR2. SAA may stimulate the expression of adhesion molecules including E-selectin through downstream signaling pathways such as NF-*κ*B and TNF-*α* via TLR2.

Our results suggest that TLR2 may have a critical role to induce adhesion molecules following TLR2 signaling and the leukocyte cascade in SAA-stimulated HAECs (Figure 6). And SAA might be a predictive risk marker for atherosclerosis onset in patients with periodontitis.

## Figures and Tables

**Table 1:** Primers used for screening SAA candidate receptors.

Gene symbol	Primer	
SELS	Sense	5′-CCC TCG ATT CAA TTG CCT TA-3′
	Antisense	5′-TGT GAC CAA TGA CCT CAT GC-3′

ABCA1	Sense	5′-TTT GCT GTA TGG GTG GTC AA-3′
	Antisense	5′-AAC AGC TCC AGC ACA AAG GT-3′

ABCA7	Sense	5′-ATG TGG TGC TCA CCT GCA TA-3′
	Antisense	5′-AAG CAG AAG TGG GGG AAG AT-3′

SCARB1	Sense	5′-CTC CCA TCC TCA CTT CCT CA-3′
	Antisense	5′-GCT CAG CTG CAG TTT CAC AG-3′

CD36	Sense	5′-GAT GTG CAA AAT CCA CAG GA-3′
	Antisense	5′-GGC TGC AGG AAA GAG ACT GT-3′

TLR2	Sense	5′-AAA TTT TGT CTG GGG TGC TG-3′
	Antisense	5′-GCA ACC AAT TCC CTT GGA TA-3′

TLR4	Sense	5′-AAT AAA CCC GGA GGC CAT TA-3′
	Antisense	5′-TCC CTT CCT CCT TTT CCC TA-3′

CST3	Sense	5′-ACC AGC CAC ATC TGA AAA GG-3′
	Antisense	5′-GGG AGG TGT GCA TAA GAG GT-3′

FPR2	Sense	5′-CTG CCA ATT CTG CTT CAC CT-3′
	Antisense	5′-GCA TCC TTG AAT GCC TCA AT-3′

AGER	Sense	5′-CTG AGG CAG GCG AGA GTA GT-3′
	Antisense	5′-TTG GCA AGG TGG GGT TAT AC-3′

GAPDH	Sense	5′-GTC AGT GGT GGA CCT GAC CT-3′
	Antisense	5′-TCG CTG TTG AAG TCA GAG GA-3′

**Table 2:** Upregulation (>5-fold) of 13 genes in HAECs after stimulation with SAA.

Gene symbol	Fold regulation
BIRC3	8.3977
CCL2	37.2715
CCL5	27.4741
CCR2	8.8766
CSF2	46.5271
FGA	51.2685
ICAM1	19.0273
IL1A	7.5685
LIF	14.3204
NFKB1	5.3147
SELE	232.3249
TNFAIP3	9.9866
VCAM1	37.2715
